# Identifying problematic drugs based on the characteristics of their targets

**DOI:** 10.3389/fphar.2015.00186

**Published:** 2015-09-01

**Authors:** Tiago J. S. Lopes, Jason E. Shoemaker, Yukiko Matsuoka, Yoshihiro Kawaoka, Hiroaki Kitano

**Affiliations:** ^1^Japan Science and Technology Agency ERATO Kawaoka Infection-Induced Host Responses ProjectMinato-ku, Japan; ^2^Department of Pathobiological Sciences, School of Veterinary Medicine, Influenza Research Institute, University of Wisconsin-MadisonMadison, WI, USA; ^3^Division of Virology, Department of Microbiology and Immunology, Institute of Medical Science, University of TokyoTokyo, Japan; ^4^The Systems Biology InstituteTokyo, Japan; ^5^Sony Computer Science Laboratories, Inc.Tokyo, Japan; ^6^Integrated Open Systems Unit, Okinawa Institute of Science and TechnologyOkinawa, Japan; ^7^Laboratory for Disease Systems Modeling, RIKEN Center for Integrative Medical SciencesYokohama, Japan

**Keywords:** multi-target drugs, drug safety, target validation, machine learning, protein networks, supervised learning

## Abstract

Identifying promising compounds during the early stages of drug development is a major challenge for both academia and the pharmaceutical industry. The difficulties are even more pronounced when we consider multi-target pharmacology, where the compounds often target more than one protein, or multiple compounds are used together. Here, we address this problem by using machine learning and network analysis to process sequence and interaction data from human proteins to identify promising compounds. We used this strategy to identify properties that make certain proteins more likely to cause harmful effects when targeted; such proteins usually have domains commonly found throughout the human proteome. Additionally, since currently marketed drugs hit multiple targets simultaneously, we combined the information from individual proteins to devise a score that quantifies the likelihood of a compound being harmful to humans. This approach enabled us to distinguish between approved and problematic drugs with an accuracy of 60–70%. Moreover, our approach can be applied as soon as candidate drugs are available, as demonstrated with predictions for more than 5000 experimental drugs. These resources are available at http://sourceforge.net/projects/psin/.

## Introduction

New compounds are traditionally discovered by using large biological screening techniques to identify substances that cause the desired effects. While this approach has been effective for years and produced the drugs used today, technological advances are shifting the drug discovery process toward a more rational approach, with computational drug-design and pathway analysis playing major roles. With the costs of compound design dramatically increasing and most of these funds being spent on drugs that never make it to market (Munos, 2009; Scannell et al., 2012), there is a clear need for new technologies to develop more specific, less toxic compounds.

Recently, *in silico* analyses have been successfully applied throughout the drug discovery pipeline. Examples include methods to help understand the changes caused by candidate compounds in protein interaction networks (Csermely et al., 2005; Yildirim et al., 2007), and algorithms to develop specific ligands that inhibit the activity of pathogen proteins (Fleishman et al., 2011; Whitehead et al., 2012). In addition, computational analyses in key studies have revealed the off-targets of drug candidates and predicted important side effects (Keiser et al., 2007, 2009; Campillos et al., 2008; Yamanishi et al., 2008; Liu et al., 2011; Lounkine et al., 2012). These studies have shown that computational analyses are an essential part of drug discovery. Yet the early identification of problematic drugs remains a major challenge.

Here, we propose a method to distinguish between compounds that are safe and those likely to be harmful. For this purpose, we considered the targets of more than 1800 approved and problematic drugs (i.e., withdrawn from market, or halted in development due to safety concerns). To study the properties of these targets, we created a protein similarity network (PSIN), in which the proteins are connected only if their sequences are similar. We found that the centrality measures of the PSIN network clearly indicated which human proteins are likely to cause harmful effects if their activities are modulated by drugs; our analysis suggested that ~5000 human proteins had characteristics that resembled those of targets of problematic drugs. Next, by using machine learning techniques, we developed an index (called the Rejection Score) to quantify the likelihood of a candidate drug being problematic. Although some substances were difficult to classify and obtained intermediate scores, most were consistent with their status of being approved or problematic.

Finally, based on the targets of more than 5000 experimental substances from major databases (~700 of which are currently undergoing pre-clinical or clinical evaluation), we predicted which drugs have a high likelihood of approval by regulatory agencies. This process of validation of individual proteins and assignment of rejection scores to candidate drugs should improve gene target selection and candidate prioritization in drug development.

## Materials and methods

### Protein sequence comparison

To perform the protein sequence comparisons required to assemble the PSIN, we used a stand-alone version of BLAST toolkit v2.2 (Camacho et al., 2009), and the human protein sequence database obtained from Uniprot (released in August 2012). We removed all splicing variants from this database, leaving only the first protein isoforms. We used the PSI-BLAST algorithm and the BLOSUM distance matrix, with a gap-open cost value of 11, gap-extension cost of 1, and minimum expectation value of 1e-03. We set the *E*-value threshold for inclusion in the multipass model at 1e-05, and six PSI-BLAST iterations or less (if the results converged before; i.e., no further sequences could be discovered in the database using the profile as the input query).

### Databases

The drugs, their targets, and status (approved, withdrawn, illicit, experimental, etc), were obtained from Drugbank (Wishart et al., 2006), from the Therapeutic Target Database (Zhu et al., 2012), and from ChEMBL (Gaulton et al., 2012). We merged all three databases into one dataset, with drugs containing targets from all three databases. While the first two databases have information about the legal status of the compounds, ChEMBL has only an indication of “therapeutic” or “non-therapeutic,” and whether a therapeutic drug contains a black-box warning. Therefore, to study the characteristics of approved and problematic drugs. we used only the drugs for which we had legal status information from DrugBank or TTD, and the therapeutic drugs from ChEMBL (Supplementary Table S1).

From the ChEMBL database, we considered only proteins targeted by a compound if they had an IC_50_-value < 30 nM. Additionally, several drugs did not have targets present in the PSIN or in the protein-protein interaction network PPI. Because these drugs targeted proteins that were isolated from the rest of the network, only drugs with at least one target present in the PSIN or PPI were considered, and for drugs with multiple targets, those targets not present in either the PSIN or the PPI were removed before any analysis was done.

For the protein-protein interaction analysis, we used HIPPIE (Human Protein-Protein Interaction Reference Schaefer et al., 2012).

### Assigning drugs approved or problematic labels

The legal status of the drugs from the two databases was highly heterogeneous, containing approved drugs (those available by prescription or over the counter), illicit, and withdrawn drugs (those removed from the market or that had their development halted due safety or efficacy concerns). For our study, we were interested in understanding what distinguishes drugs successfully used to treat patients from those causing drug attrition or those that were withdrawn from the market due harmful effects. Thus, we first identified drugs that were withdrawn from the market in several countries and classified them as problematic. Second, drugs that were discontinued during clinical trials due to safety or efficacy issues were also considered problematic. In contrast, we considered a drug “successful” if it was available for purchase. Additionally, after detailed inspection of drugs classified as “illicit” in the DrugBank database, we verified that they were mainly used to treat psychological disorders (e.g., anxiety, schizophrenia, and insomnia), and had the potential for abuse and addiction. Since these substances can be obtained in most countries when prescribed by clinicians, we concluded that most of them were not in fact illicit, but rather “controlled substances,” in which case, we also considered them approved drugs (Supplementary Table S2).

### Centralities, averages, and relevance measures

For each protein network, we calculated the betweenness of a node *v* as:

$$\begin{array}{l} B\left(v\right)=\sum \frac{sij\left(v\right)}{sij},\;with\;i\neq j,v\neq i\;and\;v\neq j \end{array}$$

where *s*_*ij*_ is the number of shortest paths between the nodes *i* and *j* and *s*_*ij*_ (*v*) is the fraction of those shortest paths passing through node *v*.

Burt's Constraint was calculated as:

$$\begin{array}{l} C\left(i\right)=\underset{j}{\sum }{\left(p_{ij}+\underset{q}{\sum }p_{iq}p_{qj}\right)}^{2},\;with\;q\neq i,j,\;and\;j\neq i \end{array}$$

where *p*_*iq*_*p*_*qj*_ is the product between the proportional strength of the node *i'*s relationship with node *q*, and the proportional strength of the node *q*'s relationship with node *j*. The details of these calculations in their original sociological context were reported by Burt (1992, 2004).

In addition, when considering multiple targets of the same drug, we transformed all individual PSIN network measures to the log10 scale and then combined their centrality measures by calculating their arithmetic means.

### Implementation, data analysis, and pre-processing

The computations involving pre-processing and machine learning classifiers were performed by using the Weka suite for data mining (Frank et al., 2004); our code was written in Java and all algorithms were used with their default parameters. The Support Vector Machine was implemented using LibSVM, with the code available at https://weka.wikispaces.com/LibSVM (visited in August 2015).

The statistical and network analyses were performed in R. Additionally, we used the iGraph package (Csardi and Nepusz, 2006) for the network analysis, the poweRlaw package for power-law fits, and the ROCR (Sing et al., 2005) package to create the ROC curves.

**Figure 1:**
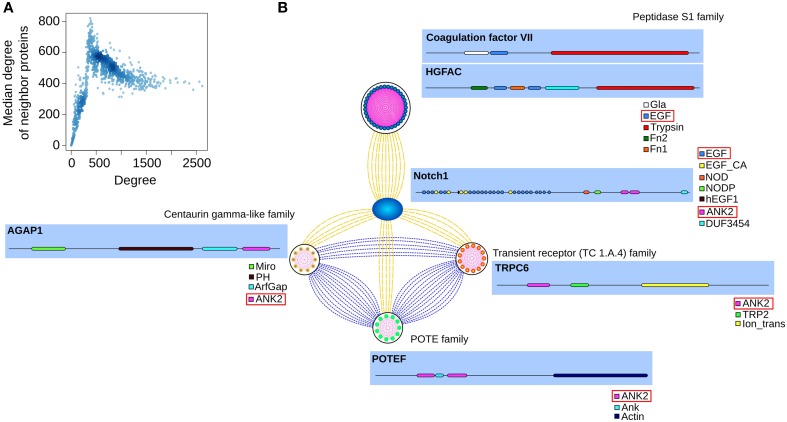
**(A)** Nodes with up to ~500 connections are neighbors of proteins with approximately the same degree. After the peak, nodes with higher degrees are connected to other nodes with ~400 connections. Darker tones of blue indicate a higher concentration of nodes with these degree values. **(B)** Depicted are a few neighbors of notch1, their families, and domain compositions (with the shared domains boxed red). In the PSIN, notch1 is connected to members of the Peptidase S1 family through their shared EGF domain. The proteins from the other three families are connected to each other and to notch1 by their ankyrin domain.

Pre-processing involved four steps: (1) not all proteins had centrality values in the PPI or in the PSIN, hence, we filled those missing values with the mean of the training and test sets separately by using the Weka function *ReplaceMissingValues*; (2) we had to over-sample the smaller class because our dataset contained more instances from the approved class than from the problematic class. Hence, we used the SMOTE (Chawla et al., 2002) algorithm for this task, with an oversample proportion of 500% and 8 nearest neighbors. We used the Tomek links (Tomek, 1976) method to remove instances whose nearest neighbors belonged to the opposite class. This strategy proved very effective relative to other pre-processing alternatives to deal with unbalanced, overlapping datasets (Batista et al., 2004). (3) we removed the instances that were on the “border” of different classes, i.e., instances that were the nearest neighbors of several instances from different classes (see Figures 1, 2 of Batista et al., 2004); and (4) we ran a preliminary cleaning step by using Multilayer Perceptron exclusively on the training set to remove the misclassified instances (we used the *RemoveMisclassified* routine from the Weka package).

For the training and testing procedure, we removed the drugs that targeted the same set of proteins. For example, if a hypothetical drug in the test set targeted proteins A, B, and C, then all other compounds in the training set that targeted A, B, and C were discarded. Additionally, we removed ~100 drugs that targeted the same proteins but had conflicting classifications (i.e., some were approved and others problematic). This ensured that we had no redundant instances in the dataset, and that the same targets were not simultaneously in the training and test sets. After calculating the mean centrality measures of all drug targets, they were scaled to the interval [0,1] by using the R package Reshape http://had.co.nz/reshape - visited in June 2015.

## Results

### Network characteristics

A protein similarity network is distinct from a protein-protein interaction network (PPI) because in the former, neighbor proteins do not necessarily interact or regulate each other's activities; instead, two proteins are connected only if their amino acid sequences are similar. Although other protein networks exist (Weston et al., 2004; Camoglu et al., 2006; Zhang and Grigorov, 2006; Atkinson et al., 2009; Rattei et al., 2010; Valavanis et al., 2010), they suffer from shortcomings such as the use of small protein datasets, employing information other than amino acid sequences, not being specific for human proteins, or not using signature-based methods to assess protein similarity. These shortcomings led us to create a network with the characteristics required to study the properties of drug targets. We used PSI-BLAST (Altschul et al., 1997) to query and compare the ~20,000 human protein sequences in the Uniprot database. BLAST searches were not reciprocal (i.e., searches with “protein A” identified “protein B” as similar, but searches with “protein B” did not necessarily identify “protein A” as similar). Therefore, to establish a link between two nodes (proteins) in the PSIN we considered only bidirectional hits.

The PSIN has ~17,000 proteins connected by ~1,700,000 edges. The network does not have a single large component; rather, it has more than 800 smaller connected components. We used the degree (the number of edges—i.e., neighbors—each node has) to quantify the connectivity of the PSIN. Its nodes have an average of 200 connections, with the most connected having ~2600 neighbors. We verified that, similar to PPI networks, the degree distribution of the PSIN also fits in the power-law distribution, where many nodes have a few connections and a few nodes have many connections (Supplementary Figure 1). Nodes with up to 500 neighbors were connected to other nodes of similar degree, and above this point, the nodes were usually connected to those with 400–500 connections (Figure 1A).

In the PSIN, the proteins are connected to each other by similarity of one or more shared domains. We verified that most of the low-degree nodes and their neighbors belonged to the same families, because these proteins had domains found only in a few other proteins. For instance, the family of alpha-defensin proteins, responsible for Gram-negative antibacterial activity, formed a cluster of only five proteins connected to each other through their exclusive and characteristic defensin domain. In contrast, highly connected proteins had a mixture of common and rare domains. For example, the notch1 protein has several repetitions of its 7 domains, which are connected to 1445 proteins from more than 50 families (Figure 1B).

In summary, the degree distribution in the PSIN resembled the power-law, like other protein networks. Low-degree proteins comprised rare domains and were mainly connected to members of the same family; high-degree proteins were composed of both rare and common domains, and were connected to members of several different families.

### Network characteristics of drug targets

By using the PSIN and a PPI database, we searched for characteristics that discriminated between the targets of approved and problematic drugs.

We obtained all drugs and their reported targets from three major drug-target databases (see Materials and Methods), and merged all three databases taking into account the different drug names and synonyms; the merged dataset contained 1802 drugs for which their legal status was available (approved, withdraw, illicit, etc., Supplementary Table S1), and more than 5000 experimental drugs (Supplementary Table S2). Next, we assigned a simplified class label to each of the 1802 drugs, indicating whether the drug was considered safe and was marketed (approved), or if it had had its development halted or was withdrawn from the market (problematic).

**Figure 2:**
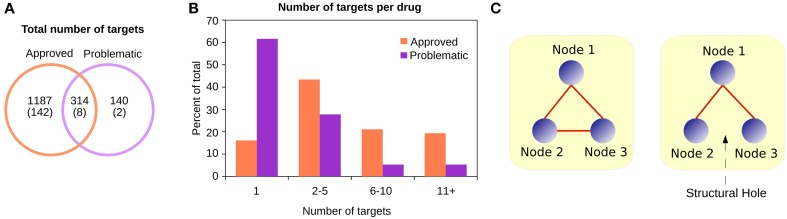
**(A)** Although most targets of approved drugs are exclusive, the problematic targets are almost entirely covered by the approved category. Between parentheses are the number of singleton proteins in the PSIN. **(B)** Approved and problematic drugs have different numbers of reported targets. While most problematic drugs have only one target reported, approved drugs have several—identified either by the community after the drug is marketed or by companies as part of the drug-approval process. **(C)** The Burt's constraint was proposed in a sociological context to study positions of advantage for individuals in a group. In this simple example, if the nodes are individuals, on the left no node can negotiate or bargain with the others, since they all have alternative connections. However, on the right, if a structural hole exists, Node 1 is in a better position, since the other two nodes may not be aware of each other's existence;hence, Node 1 is less “constrained” than the other two. In a protein similarity context, proteins with low constraint values are generally those with several common domains, located between different protein families. In contrast, proteins with large constraint values are the peripheral nodes, with a few domains shared among only a few other proteins.

We observed that the targets of approved and problematic drugs largely overlapped (Figure 2A), and there were more reported targets in the combined databases for the approved drugs than for the problematic drugs (Figure 2B). This is due to the strict requirements for drug approval by regulatory agencies, since before going to market, companies must provide detailed reports about modes of action, and after a compound is released, researchers from academia often report additional targets.

For each target of the approved and problematic drugs, in addition to the degree (the number of neighbors a protein has in the network), we calculated their betweenness, closeness centrality, and Burt's constraint in the PSIN and PPI networks. The betweenness describes how central a node is, counting the number of shortest paths that must pass through that node to connect the other nodes in the network. The closeness centrality from a node measures how many steps are necessary to reach every other node. The Burt's constraint, was first employed in a socio-psychological context, where the author studied the location of individuals in a large social network and quantified which individuals are in a position of advantage, located between groups, and have access to information and resources from different environments (Burt, 1992, 2004) (Figure 2C).

**Figure 3:**
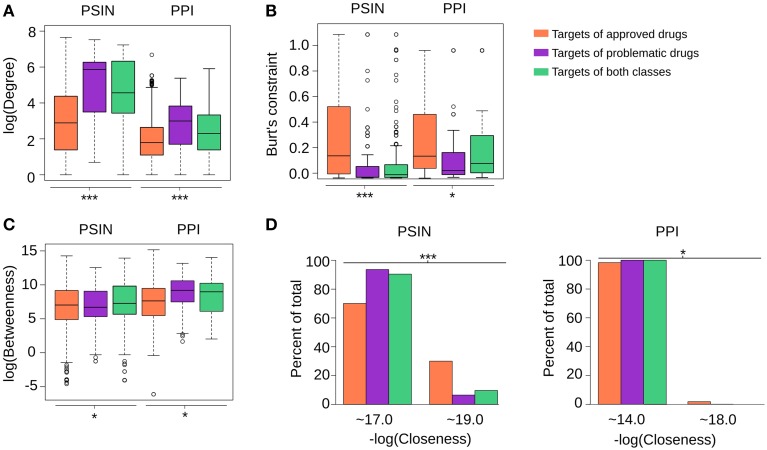
**(A–D)** In general, targets of problematic drugs have high degrees and closeness centralities in the PSIN and PPI networks. However, their betweenness values are not significantly different from the targets of approved drugs in either protein network (One-Way ANOVA, ^***^p < < 0.0001 and ^*^p>0.05, sample sizes for each group are the same as depicted in Figure 2A). The closeness from the targets of both networks was close to two main values, differing by only decimal digits; therefore, we rounded the values to their closest integer, namely 17 or 19 in the PSIN and 14 or 18 in the PPI. While three PSIN centrality measures were found to be strong indicators of the differences between targets of problematic and approved drugs, the centrality measures of the PPI network could also detect these differences, albeit in a moderate fashion (Tukey's Honest Significance Difference—Supplementary Figure 2). Overall, this likely stems from the fact that the current PPIs still have only ~10,000 proteins and numerous false-positive interactions; with new proteins and high-quality interactions being constantly added, we expect this to change in the future.

Compared with proteins targeted by the approved drugs, those targeted by problematic compounds had a significantly higher degree in both networks, and much lower closeness centrality and Burt's constraint values (Figure 3; for each centrality measure, One-Way ANOVA, *p* < 0.0001, followed by Tukey's HSD test; Supplementary Figure 2). In contrast, we observed no significant differences in the betweenness values of proteins targeted by problematic and those targeted by approved drugs in the PSIN or in the PPI network. These findings indicate that while targets of approved drugs have protein domains that are not shared among many other proteins and are involved in fewer interactions, targets of problematic drugs have domains that are more common throughout the proteome and have more protein interactions reported.

Figure 3 suggests that proteins targeted by approved compounds and problematic compounds have characteristics similar to problematic targets. This finding could shed light on why some drugs are approved, while others are rejected, even though they target the same protein. For example, VEGF receptors, which are involved in blood vessel growth, are popular anti-cancer targets. Usually, the drugs targeting these receptors cause major side effects (Roodhart et al., 2008) including hypertension, coagulation disorders, and neurotoxicity. We found that the three VEGF receptors had characteristics similar to other targets of problematic drugs, suggesting that compounds that target proteins with these characteristics will either be unapproved or, if approved, will likely cause harmful effects.

Next, we asked how many proteins share the characteristics of the approved and problematic targets. We observed that ~65% of problematic drug-targets had a degree value > 110 and a Burt's constraint <0.025, whereas only ~22% of approved drug-targets had these characteristics. In the PPI, ~62% of problematic targets had a degree value >11 and a Burt's constraint <0.1, compared with ~33% of approved drug-targets with these characteristics. In general, these values are ones that show the highest separation between the targets of problematic and approved drugs (Supplementary Figure 3). When we consider the centrality measures separately, in the PSIN, ~7600 proteins had degree and Burt's constraint values similar to those of problematic targets, and in the PPI, ~4600 had degree and constraint values similar to other proteins of the problematic group. When we consider the characteristics of both networks together, ~1200 proteins had measures that closely resembled those of problematic drug targets. Notably, this group has several popular targets of anticancer drugs, including almost all members of the cyclin-dependent kinase family, aurora kinases, and Pim/PLK serine threonine kinases. We then attempted to verify whether compounds targeted neighbor proteins in the PSIN. We built a contingency table counting the number of compounds that targeted neighbor proteins and the number of compounds that did not target neighbor proteins in the PSIN and tested its statistical significance by using Fisher's exact test. We obtained a *p*>0.05, confirming that compounds often target proteins with no detectable sequence similarity (Keiser et al., 2007; Apsel et al., 2008; Yamanishi et al., 2008).

Taken together, our results indicate that the centrality measures calculated from the PSIN (and to a lesser extent from the PPI) can be used to distinguish between individual targets of approved and problematic drugs. These characteristics define the “danger zone” for therapeutic modulation, that is, they serve as indictors that modulating the activity of these proteins may be harmful to human health.

### Classifying multi-target drugs

Since drug targets of approved and problematic drugs could be distinguished individually, we asked whether evaluating the characteristics of all proteins targeted by a compound would help to predict the compound's safety and consequently, its approval or rejection. For classification and prediction tasks, supervised learning algorithms use a training set with examples assigned to different classes, and after a training phase, these algorithms attempt to predict the classes of instances they have not seen before. In our case, our training set comprised drugs, their targets, and their status (approved or problematic).

We built a dataset by calculating the centrality measures used above for each target of each drug; however, since most drugs have multiple targets, we combined the centrality measures of these targets using the means of their individual measures (see Materials and Methods). Overall, our dataset comprised 1802 drugs: 1445 approved and 357 problematic (Supplementary Table S1). As in most real-life scenarios, this dataset is characterized by the imbalance between the number of approved vs. problematic drugs–a characteristic that is notably difficult for machine learning algorithms (Batista et al., 2004). Therefore, we pre-processed the dataset to increase the sensitivity of the classifiers to the characteristics of the problematic class (see Materials and Methods), and for the classification routine, we compared the performance of 14 machine learning classifiers, namely KStar (Cleary and Trigg, 1995), Naive Bayes (John and Langley, 1995), J48 (Quinlan, 1993), Thresold Selector (Witten et al., 2011), Multilayer Perceptron (MLP)(Bishop, 1995), JRip (Cohen, 1995), IB1 (Aha et al., 1991), PART (Frank and Witten, 1998), END (Dong et al., 2005), Random Tree (Breiman, 2001), Rotation Forest (Rodríguez and Kuncheva, 2006), Random Forest (Breiman, 2001), Decorate (Melville and Mooney, 2003), and Support Vector Machines (SVM)(Cortes and Vapnik, 1995).

We asked how the classifiers perform if we use only the centrality measures from the PSIN, from the PPI, or a combination of both. We divided the input dataset into 70% of instances for training and 30% for prediction, with no overlapping drugs between them. Drugs that bind the same set of protein targets were removed to prevent obvious redundancies during classifier evaluation (see Materials and Methods). This procedure was repeated 100 times to quantify the prediction accuracy for each set of centrality measures. We then verified that although the topological characteristics of the PPI network could moderately distinguish between individual approved and problematic drugs, the predictive power of the machine learning algorithms was highest when using only the centrality measures of the PSIN network (Supplementary Figure 4), therefore, for subsequent analyses we used only the PSIN.

After close inspection of the drug-targets dataset, we realized that confounding factors of the drug-binding protein data might affect classifier performance (e.g., differing numbers of binding partners per drug, missing drug-targets in the protein networks). Therefore, we designed three tests to determine whether the PSIN data could enhance classifier performance over the performance obtained when only these confounding factors were considered. In the first test, we shuffled the class-labels (i.e., approved and problematic labels and the targets of all drugs—always keeping the same proportions as the original datasets—and compared them to the performances obtained when using the standard dataset, by using the 70%–30% division for training and testing sets, respectively. We verified that while most classifiers had an area under the ROC curve (AUC) close to 0.7 when trained to the complete dataset, the classifiers generally performed close to random guessing (AUC~0.5) when trained using random datasets (*p* < 0.01, comparing the AUCs of each classifier, Wilcoxon two-sided signed-rank test).

In the second test, we developed a more stringent procedure wherein we created 40 randomized datasets also by shuffling the labels of the proteins in the PSIN, but here, for each single cross-validation, we first randomly split the standard dataset (i.e., that derived from the PSIN) into the 70%–30% training–test sets and determined the AUC. Next, we took each of the 40 randomized datasets and carefully divided them into training and testing data, making sure that we selected the same drugs that were used for training and testing with the standard data, and calculated the AUC. The 40 AUCs from the randomized data represented our null distribution, that is, the expected AUC achieved when drugs can bind any proteins. From this null distribution, we determined the likelihood that the AUC achieved by the standard data happened by chance, by comparing all runs of the randomized data to the standard dataset, and we verified that the overall classification procedure had better performance than random datasets in more than 88% of all comparisons (Supplementary Figure 5).

In the final test, we shuffled only the PSIN protein labels, removing the class distinctions discussed in Figure 3, while keeping the same network topology distributions (i.e., the power-law degree distribution). Again, we divided the dataset into 70%–30% for training and testing, respectively, repeating this procedure 100 times, and compared the results to the standard dataset. We observed that the classifiers performed considerably better than all of the shuffled networks (Supplementary Figure 6).

Together, these results demonstrate that the PSIN and the machine learning classifiers can overcome the effects of confounding factors, and distinguish multi-target problematic and approved drugs based solely on the network characteristics of their targets. Some of the algorithms outputted only binary classifications (END, Random Tree, SVM), or had the same underlying base classifier (J48); therefore, for further classifications, we used three algorithms (KStar, MLP, and Rotation forest) that were built using different underlying principles and outputted a probability that a drug belonged to the problematic class. This approach should compensate for any inevitable biases that all algorithms have.

### Predicting drug safety

After analyzing the capabilities of the classifiers, we used them as a prediction tool for new multi-target drugs. For fairness, all previous tests to study the characteristics of the classifiers had been performed without parameter or dataset optimization. However, to use as a prediction tool, it is desirable to fine-tuned the input dataset and include only the most meaningful examples in the training set.

While the approved drugs are generally compounds approved by regulatory agencies and successfully commercialized, the problematic drugs were deemed problematic for one or more of 10 different reasons (Supplementary Table S3). We wanted to test whether individually removing each of these 10 reasons from the input set would improve the classification.

For this purpose, we created 10 different datasets containing all of the approved and problematic drugs except those in each of the 10 groups that led to the drug failure. Each of these 10 datasets was then randomly divided into training and test sets by using the 70%–30% proportion, and the AUC of the classification was calculated. This procedure was repeated 100 times and our results showed that removal of one group of problematic drugs (those deemed “withdrawn”) considerably improved the classification (*p* < 0.001, One-Way ANOVA, followed by Tukey's HSD test). Most likely, these compounds targeted proteins that were also targeted by approved drugs, but—in contrast to approved drugs—had harmful effects. Thus, by removing these drugs from the dataset, we removed a confounding factor and consequently reduced the false positives and increased the overall drug classification.

Next, from the complete dataset (with 1802 drugs), we selected one drug at a time and used the drugs from the optimized dataset as a training set. Importantly, we also removed from the training set any drug that targeted the same proteins as the drug being evaluated (the predictions are available in Supplementary Table S4).

We found that the classification of existing and experimental drugs into two classes (i.e., approved and problematic) could be over-simplistic; for drug development, it is more informative to quantify how likely a compound is to cause harm (Evans et al., 2009). Therefore, we created an index, which we named the “Rejection Score” (RS), by using the average of the probabilities calculated by use of the three chosen classifiers. We used this index to indicate whether a compound was predicted to be safe (RS close to 0.0) or more likely to be toxic (RS close to 1.0).

**Figure 4:**
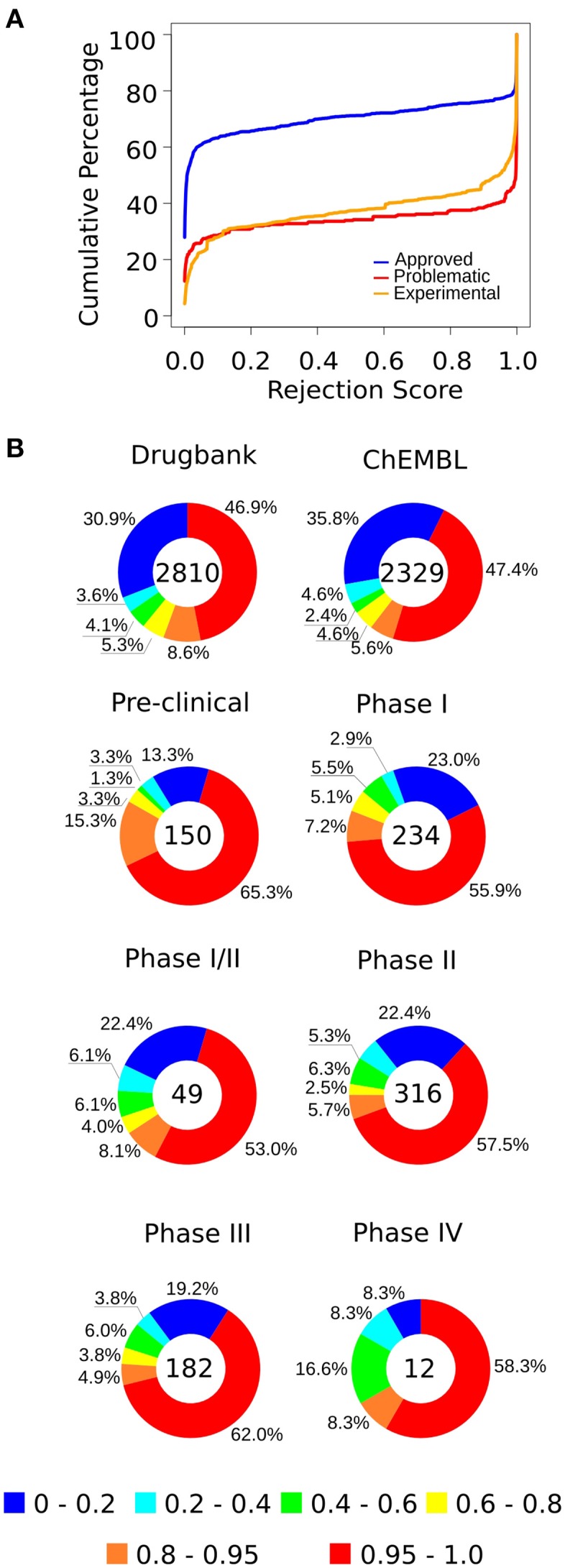
**(A)** The cumulative percentage of approved, experimental, and problematic drugs, according to their rejection scores (RSs) (the complete predictions are available in Supplementary Tables S3, S4). **(B)** We predicted the status of experimental drugs from the TTD, Drugbank, and ChEMBL databases. In general, more than half of the drugs have high rejection scores, whereas about 20–30% have RSs that position them among the low-risk compounds. Each chart contains the number of drugs of the respective group.

We found that 55% of approved drugs had a RS <0.02 (Figure 4A, Table 1); yet, only 23% of problematic drugs had RSs close to 0.02. Conversely, 23% of approved drugs and 61% of problematic compounds had RSs greater than 0.9. Beyond this point, we observe a sharp increase in the number of problematic drugs and a slow increase in the number of approved drugs; hence, drugs with RSs of 0.9–0.95 could be considered “high-risk” compounds that are likely to cause strong side effects (nonetheless, their use may be warranted to treat life-threatening diseases). It is important to note that these cut-offs are arbitrary and may vary depending on the risk that is deemed acceptable. Moreover, given that drugs have distinct numbers of targets, we tested the rejection score and observed that it moderately correlated with its number of targets (Supplementary Figure 7).

**Table 1:** **Percentage of drugs classified according to their Rejection Score**.

**Rejection score**	**Percentage of drugs (%)^a^**	
	**Approved**	**Problematic**
0.0004	88.9	1.96
0.0008	76.2	10.36
0.0055	53.28	19.04
0.2400	33.14	30.53
0.9291	23.73	40.05
0.9998	6.92	85.15

a *In this dataset, 357 drugs were deemed “Problematic”; 1445 were deemed “Approved.” See Supplementary Table S4 for details*.

Next, we asked whether the Rejection Scores reflected the known adverse reactions of marketed drugs. To answer this question, we analyzed the labels and package inserts of 245 drugs obtained from the SIDER database (Kuhn et al., 2010). We chose drugs with RSs covering the full range of predicted scores (i.e., from 0.0 to 1.0); for all selected drugs, we listed the indicated precautions, contraindications, adverse reactions, warnings, and where available, boxed warnings (Supplementary Table S5).

Several drugs with an RS of 0.9–1.0 had associated warnings and cautions regarding the risk of severe reactions, for example, beta-blocker drugs (Pirbuterol, Atenolol, Alvimopan), which can cause life-threatening reactions including heart failure, bradycardia, and angina. The side effects of beta-blockers have been known for decades (Frishman, 1988), and the high RSs of these drugs likely stem from the fact that their target proteins (Beta-1 and Beta-2 adrenergic receptors) have characteristics similar to those of other problematic drug targets, namely a high-degree (500+), and a low Burt's constraint (<0.006).

At the other end of the scale, we found that drugs with RSs of 0.0–0.2 and of 0.2–0.4 have been commercially available for years; an example is Bumetanide (RS 0.26), a diuretic that may cause profound water loss and electrolyte depletion only if used in excess. Bumetanide's reported targets include proteins involved in the transport of potassium, chloride, and sodium, and these transporters have low PSIN degree values (~24) and high Burt's constraints (~0.14), which are characteristics of proteins that are relatively isolated in the network and that are similar to only a few others. Another example is Diazepam (RS 0.0005), a benzodiazepine broadly prescribed since 1963 to treat anxiety and insomnia that can cause unpleasant, but manageable side effects, such as nausea, skin rashes, and headache (Riss et al., 2008). The targets of Diazepam, gamma-aminobutyric acid (GABA) receptors, have low PSIN degree values (46), and a high Burt's constraint (0.0826), and form a cluster in which these proteins connect to each other and to a few other GABA receptor subtypes.

In addition to drugs classified as approved or problematic, we also calculated the RS of ~5000 experimental drugs. Experimental drugs listed in TTD and Drugbank are those currently being evaluated in clinical and pre-clinical trials, that have already undergone such evaluation, did not make it to market, or are being tested for alternative indications. We also predicted the RSs of non-therapeutic substances from ChEMBL. The respective compounds selected from all three databases are listed in Supplementary Table S3, and their RS predictions are shown in Supplementary Table S6.

For experimental drugs, while more than 50% of compounds were predicted to have high rejection scores (>0.95), only 15–20% of compounds in clinical trials had scores similar to those of approved drugs (Figure 4B), suggesting that only a small number of these candidate compounds may be approved and not cause severe side effects. These observations are consistent with the known high attrition rates observed in the pharmaceutical industry (Kola and Landis, 2004).

To summarize, most of our predictions seem to match the status of marketed drugs and their reported adverse reactions, and in general, our results show that drugs with high RSs were more likely to be discontinued in their development or be withdrawn from the market after commercialization.

## Discussion

Here, we investigated the characteristics of proteins targeted by approved and problematic drugs. We found that they have distinguishing characteristics that can be readily identified by using protein similarity and protein-protein interaction networks. In addition, we used machine learning methods to devise a score to quantify the risks of a drug being harmful to human health. We used this approach to predict the safety of several drugs and found that, for the most part, the prediction is consistent with their status of approved or problematic.

Given the prediction accuracy of ~70%, it is unlikely that pharmaceutical companies would use such prediction results for Go/No-Go decision on compounds that may be already in the late pre-clinical or clinical stages. However, such information may be highly valuable in creating an overall portfolio of the candidate compounds from their very large chemical libraries. If a pharmaceutical company uses our method to evaluate millions of compounds and decides to moderately bias their choices according to the ratings presented here, the long-term outcome may be very different. This will be increasingly important when assessing the suitability of multiple-target drugs—i.e., it is essential to efficiently identify combinations of targets and compounds that are both safe and effective.

Interestingly, while attempting to find general properties of individual proteins that may be associated with side effects, we found characteristics from protein networks that clearly distinguish between targets of approved and problematic drugs (Figure 3). The degree, betweenness, and closeness centrality of proteins are well known measures and are broadly used in network studies. The Burt's constraint is already used in sociological studies and interestingly, revealed itself as a strong indicator of protein druggability (Figure 3B). Together, these network characteristics define a new axis along which drug targets can be assessed for their viability. In addition, together with other considerations (e.g., existence of accessible binding pockets, and the location and time-point of expression), this methodology can help validate safe targets whose modulation is therapeutically relevant (Bunnage, 2011). Further, the correlation between the RSs and the severity of the reported adverse reactions was not perfect; some drugs with a high RS had only mild adverse reactions, while others with low RSs had clear warnings about their potential harm. A potential explanation is that some drugs targeting highly connected proteins were approved despite their known adverse reactions (e.g., anti-cancer drugs), while relatively safe drugs may not have been developed due to business decisions. Thus, the Rejection Score should not be considered in isolation, but in conjunction with the network centralities of the drug's targets. For instance, a drug with low RS targeting high-degree proteins suggests that this compound is likely to cause moderate or severe side effects but still has the potential to be approved, depending on its indication. Conversely, if a compound has a high RS and targets high-degree proteins, this is a strong indication that this compound will be problematic because only a few or no other drugs targeting proteins with similar characteristics have been successfully commercialized.

When developing therapeutic compounds it is difficult to assess beforehand which proteins can be targeted without causing major side effects but still overcome cell tolerance to perturbations (Kitano, 2007; Hopkins, 2008). Moreover, understanding and anticipating side effects can be complex. For instance, the weight gain, diabetes, and cardiovascular problems experienced by patients being treated with antipsychotic drugs have causes that remain unclear (De Hert et al., 2012), although mounting evidence suggests that a compound's side effects are caused by modulation of its primary and secondary targets (Xie et al., 2009; Correll et al., 2011; Lounkine et al., 2012).

Some cases were difficult to classify. A notable example was Thalidomide (*RS* = 0.0004), a drug used to treat morning sickness and removed from the market after it was associated with birth defects (Stephens et al., 2000). Due to its inhibition of blood vessel growth (D'Amato et al., 1994), this compound was investigated to treat cancer (Verheul et al., 1999) and appeared to improve the survival of multiple myeloma patients (Singhal et al., 1999). With a low RS, this drug targeted proteins with characteristics of both approved and problematic targets, indicating that in addition to network characteristics, it is essential to verify that the stage of development and the location for target inhibition is appropriate (i.e., inhibiting angiogenesis in tumors is desirable, but during limb development is catastrophic).

Naturally, our approach has limitations. First, there are false-positives in both the PPI and PSIN networks. In the former, false-positives exist mainly due different experimental setups (Venkatesan et al., 2009), and in the latter, the algorithm used to build the network has a statistical cut-off, which, although strict, does not completely prevent proteins from being set as neighbors by chance. Second, the list of targets reported for each drug is incomplete and it varies from reporting several targets for approved drugs, to only a few for problematic compounds. As recently demonstrated, known drugs have multiple protein off-targets, bound with enough specificity to interfere with their functions (Campillos et al., 2008; Yamanishi et al., 2008; Keiser et al., 2009; Lounkine et al., 2012). Additionally, the targets reported in the database might show not only direct interaction, but also indirect activation or repression. Therefore, future studies should take into account the nature of the interaction between the compound and the members of pathways, as well as which isoforms of the proteins are the actual targets of the drugs (here, as in most pathway databases and protein networks, we considered only the first splicing variant). Another limitation of the approach presented here is the mismatch between the RSs of drugs and their reported adverse effects. The reason for this mismatch is known, but its resolution is markedly complicated. Drugs approved despite their known severe adverse effects, as well as drugs deemed problematic for business-related reasons, are confounding factors even for the most sophisticated classifiers. Ideally, one would separate drugs by indication or class, train classifiers using only the drugs in each group, and finally check the predictions against drug labels and the complete documentation for the drugs in the training set. However, this is not practical at present because no database has enough positive and negative examples from each drug class, and only a fraction of all results and adverse reactions observed during clinical trials are available at present (Wieseler et al., 2013). Therefore, to benefit from the methods presented here, it is important to always consider the RS of drug candidates together with the centrality of the protein targets. Nevertheless, the quality of protein networks and of the drug-target databases is constantly improving, and despite these limitations, the methods presented here predicted the safety or danger of 60–70% of known drugs (Figure 4A). Moreover, we believe that as more comprehensive post-translational and structural information becomes available, its integration to the PSIN will enhance its predictive capabilities, furthering our understanding of the mechanisms of drug action and its effects on protein structures.

Finally, our prediction that several experimental drugs may not be approved provides evidence that the approaches presented here can be used long before a drug reaches the end of the development pipeline (Scannell et al., 2012); in fact, as soon as the targets of a compound are determined, we would recommend that that compound be subjected to the procedures described here.

## Author contributions

TL designed the study, implemented the algorithms, analyzed the data and wrote the manuscript with feedback from all of the authors; JS helped elaborate the tests, implemented part of the algorithms and analyzed the data; YM helped conceive the study and analyzed the data; HK and YK provided the funding and coordinated the research efforts.

## Funding

This work was supported by the Japanese Science and Technology Agency, through the ERATO Kawaoka influenza host-response project.

### Conflict of interest statement

Tiago J. S. Lopes, Yoshihiro Kawaoka, and Hiroaki Kitano have a patent application for the drug classification approach presented in this manuscript. Jason E. Shoemaker and Yukiko Matsuoka declare that the research was conducted in the absence of any commercial or financial relationships that could be construed as a potential conflict of interest.

## Supplementary material

Supplementary Figure S1 **We verified that the PSIN degree distribution could be fitted in the power-law**. The figure depicts the degree distribution (black circles) and the curve-fit (in red), with the modifying exponent parameter indicated in the figure. The fit is satisfactory for most nodes, but those with a higher degree have some deviation from the fitted curve. Additionally, we can observe the degree distribution for the HIPPIE database, and for a sequence of 15,000 random numbers chosen with replacement from the interval [1, 2000].

Supplementary Figure S2 **Tukey's Honest Significant Difference test indicating the differences between the groups of drug targets**. APP stands for Approved drug-targets, PRO Problematic drug-targets, and BOTH are the proteins targeted by drugs from both groups. The sample sizes of each group are the same as those shown in Figure 2A.

Supplementary Figure S3 **The figure shows the distribution of the degree and the Burt's constraint of the targets of approved and problematic drugs**. In the PSIN, the highest separation between targets of approved and problematic drugs occurs at a degree value of 110; 75–80% of approved drug targets have values lower than this value, whereas only 30–35% of problematic drug targets have degree values smaller than 110. Additionally, we can observe that the separation of targets of problematic and approved drugs is much narrower in the PPI than in the PSIN, making it more difficult to distinguish the potential therapeutic targets based on the centrality measures of the PPI network.

Supplementary Figure S4 **Boxplots with popular measures used to evaluate classifiers and different datasets**. We can verify that for most measures and classifiers, the PSIN alone yields the best results.

Supplementary Figure S5 **We created randomized datasets that maintained the dependencies between the samples by shuffling the labels of the proteins in the PSIN and recalculating the average network topologies for each drug to create a single randomized dataset**. In total, we created 40 randomized datasets. **(A)** Depicts an example, for a single cross-validation where we randomly split the standard dataset (i.e., that derived from the PSIN) into a training set and a test set and determined the AUC. Next, we took each of the 40 randomized datasets, carefully split them into training and testing data making sure we selected the same drugs that were used for training and testing with the standard data, and calculated the AUC. The 40 AUCs from the randomized data represent our null distribution, that is, the expected AUC achieved when drugs can bind any proteins. From this null distribution, we determined the likelihood that the AUC achieved by the standard data happened by chance. Thus, the *p*-value for a single iteration is the fraction of AUCs from the randomized data that was greater than or equal to the AUC achieved with the standard data; for example, a *p*-value of 0.1 would indicate that in 10% of cases the randomized data yielded an AUC equivalent to the standard run **(B)**, Dataset #0 is the standard run, the remaining are the randomized datasets. We then repeated this for 120 cross-validation iterations (totaling 4920 calculations) and calculated the average and the median *p*-values observed across all iterations of the three classifiers used to formulate the Rejection Score. **(C)** Depicts histograms of the *p*-values achieved from 120 cross-validation iterations.

Supplementary Figure S6 **Depicted are the Area Under the ROC Curves (AUCs) where the Complete Dataset (i.e., original PSIN), is compared with a dataset in which we kept the networks intact, but shuffled the protein labels**. We observe that most classifiers obtained higher AUCs than the shuffled datasets, indicating that even though confounding factors (e.g., number of targets per drug) play a role in the classification, the classifiers were still able to provide significant added value to the overall procedure. In each panel, the groups were compared by using the Wilcoxon two-sided signed-rank test, and in all cases, they were distinct with *p* < < 0.01.

Supplementary Figure S7 **We observed a negative correlation (−0.35) between the number of targets a drug has and its rejection score (if the number of targets was in the logarithmic scale, the correlation was −0.51), yet, most drugs with only a few or with many targets still had similar rejection scores**. The figure also suggests that we were not capturing the characteristics of only one or two of the databases.

Supplementary Table 1 **Drugs, targets, and their regulatory status**.

Supplementary Table 2 **Experimental drugs and their targets**.

Supplementary Table 3 **Criteria used for the drug categorization**.

Supplementary Table 4 **Computational classification of marketed drugs**.

Supplementary Table 5 **Drugs, Rejection Scores, and reported adverse effects**.

Supplementary Table 6 **Computational classification of experimental drugs**.

## References

[B1] Aha D. W. Kibler D. Albert M. K. (1991). Instance-based learning algorithms. Mach. Learn. 6, 37–66. 10.1007/BF0015375925928060

[B2] Altschul S. F. Madden T. L. Schäffer A. A. Zhang J. Zhang Z. Miller W.. (1997). Gapped BLAST and PSI-BLAST: a new generation of protein database search programs. Nucleic Acids Res. 25, 3389–3402. 10.1093/nar/25.17.3389 9254694 PMC146917

[B3] Apsel B. Blair J. A. Gonzalez B. Nazif T. M. Feldman M. E. Aizenstein B.. (2008). Targeted polypharmacology: discovery of dual inhibitors of tyrosine and phosphoinositide kinases. Nat. Chem. Biol. 4, 691–699. 10.1038/nchembio.117 18849971 PMC2880455

[B4] Atkinson H. J. Morris J. H. Ferrin T. E. Babbitt P. C. (2009). Using sequence similarity networks for visualization of relationships across diverse protein superfamilies. PLoS ONE 4:e4345. 10.1371/journal.pone.0004345 19190775 PMC2631154

[B5] Batista G. E. A. P. A. Monard M. C. Bazzan A. L. C. (2004). Improving rule induction precision for automated annotation by balancing skewed data sets. Knowl. Explor. Life Sci. Inform. Proc. 3303, 20–32. 10.1007/978-3-540-30478-4_3

[B6] Bishop C. M. (1995). Neural Networks for Pattern Recognition. Oxford; New York, NY: Clarendon Press; Oxford University Press.

[B7] Breiman L. (2001). Random forests. Mach. Learn. 45, 5–32. 10.1023/A:101093340432426311752

[B8] Bunnage M. E. (2011). Getting pharmaceutical RandD back on target. Nat. Chem. Biol. 7, 335–339. 10.1038/nchembio.581 21587251

[B9] Burt R. S. (1992). Structural Holes: The Social Structure of Competition. Cambridge, MA: Harvard University Press.

[B10] Burt R. S. (2004). Structural holes and good ideas. Am. J. Soc. 110, 349–399. 10.1086/421787

[B11] Camacho C. Coulouris G. Avagyan V. Ma N. Papadopoulos J. Bealer K.. (2009). BLAST+: architecture and applications. BMC Bioinformatics 10:421. 10.1186/1471-2105-10-421 20003500 PMC2803857

[B12] Camoglu O. Can T. Singh A. K. (2006). Integrating multi-attribute similarity networks for robust representation of the protein space. Bioinformatics 22, 1585–1592. 10.1093/bioinformatics/btl130 16595556

[B13] Campillos M. Kuhn M. Gavin A. C. Jensen L. J. Bork P. (2008). Drug target identification using side-effect similarity. Science 321, 263–266. 10.1126/science.1158140 18621671

[B14] Chawla N. V. Bowyer K. W. Hall L. O. Kegelmeyer W. P. (2002). SMOTE: Synthetic minority over-sampling technique. J. Artif. Intell. Res. 16, 321–357. 10.1613/jair.95326129418

[B15] Cleary J. Trigg L. (1995). K^*^: an instance-based learner using an entropic distance measure, in Proceedings of the 12th International Conference on Machine Learning (Morgan Kaufmann), 108–114.

[B16] Cohen W. (1995). Fast effective rule induction, in Proceedings of the Twelfth International Conference on Machine Learning (Morgan Kaufmann), 115–123.

[B17] Correll C. U. Lencz T. Malhotra A. K. (2011). Antipsychotic drugs and obesity. Trends Mol. Med. 17, 97–107. 10.1016/j.molmed.2010.10.010 21185230 PMC3053585

[B18] Cortes C. Vapnik V. (1995). Support-vector networks. Mach. Learn. 20, 273–297. 10.1007/BF0099401817306960

[B19] Csardi G. Nepusz T. (2006). The Igraph software package for complex network research. InterJournal Complex Systems, 1695. Available online at: http://www.igraph.org

[B20] Csermely P. Agoston V. Pongor S. (2005). The efficiency of multi-target drugs: the network approach might help drug design. Trends Pharmacol. Sci. 26, 178–182. 10.1016/j.tips.2005.02.007 15808341

[B21] D'Amato R. J. Loughnan M. S. Flynn E. Folkman J. (1994). Thalidomide is an inhibitor of angiogenesis. Proc. Natl. Acad. Sci. U.S.A. 91, 4082–4085. 10.1073/pnas.91.9.4082 7513432 PMC43727

[B22] De Hert M. Detraux J. Van Winkel R. Yu W. Correll C. U. (2012). Metabolic and cardiovascular adverse effects associated with antipsychotic drugs. Nat. Rev. Endocrinol. 8, 114–126. 10.1038/nrendo.2011.156 22009159

[B23] Dong L. Frank E. Kramer S. (2005). Ensembles of balanced nested dichotomies for multi-class problems, in Proceedings of the 9th European conference on Principles and Practice of Knowledge Discovery in Databases (Porto: Springer-Verlag).

[B24] Evans R. Hinds S. Hammock D. (2009). Portfolio analysis and RandD decision making. Nat. Rev. Drug Discov. 8, 189–190. 10.1038/nrd2744 19182818

[B25] Fleishman S. J. Whitehead T. A. Ekiert D. C. Dreyfus C. Corn J. E. Strauch E. M.. (2011). Computational design of proteins targeting the conserved stem region of influenza hemagglutinin. Science 332, 816–821. 10.1126/science.1202617 21566186 PMC3164876

[B26] Frank E. Hall M. Trigg L. Holmes G. Witten I. H. (2004). Data mining in bioinformatics using Weka. Bioinformatics 20, 2479–2481. 10.1093/bioinformatics/bth261 15073010

[B27] Frank E. Witten I. H. (1998). Generating accurate rule sets without global optimization, in Proceedings of the Fifteenth International Conference on Machine Learning (San Francisco, CA: Morgan Kaufmann Publishers Inc), 144–151.

[B28] Frishman W. H. (1988). Beta-adrenergic receptor blockers. Adverse effects and drug interactions. Hypertension 11, II21–II29. 2895072 10.1161/01.hyp.11.3_pt_2.ii21

[B29] Gaulton A. Bellis L. J. Bento A. P. Chambers J. Davies M. Hersey A.. (2012). ChEMBL: a large-scale bioactivity database for drug discovery. Nucleic Acids Res. 40, D1100–D1107. 10.1093/nar/gkr777 21948594 PMC3245175

[B30] Hopkins A. L. (2008). Network pharmacology: the next paradigm in drug discovery. Nat. Chem. Biol. 4, 682–690. 10.1038/nchembio.118 18936753

[B31] John G.H. Langley P. (1995). Estimating continuous distributions in Bayesian classifiers, in Proceedings of the Eleventh Conference on Uncertainty in Artificial Intelligence (Montreal, QC: Morgan Kaufmann Publishers Inc.).

[B32] Keiser M. J. Roth B. L. Armbruster B. N. Ernsberger P. Irwin J. J. Shoichet B. K. (2007). Relating protein pharmacology by ligand chemistry. Nat. Biotechnol. 25, 197–206. 10.1038/nbt1284 17287757

[B33] Keiser M. J. Setola V. Irwin J. J. Laggner C. Abbas A. I. Hufeisen S. J.. (2009). Predicting new molecular targets for known drugs. Nature 462, 175–181. 10.1038/nature08506 19881490 PMC2784146

[B34] Kitano H. (2007). Towards a theory of biological robustness. Mol. Syst. Biol. 3:137. 10.1038/msb4100179 17882156 PMC2013924

[B35] Kola I. Landis J. (2004). Can the pharmaceutical industry reduce attrition rates? Nat. Rev. Drug Discov. 3, 711–716. 10.1038/nrd1470 15286737

[B36] Kuhn M. Campillos M. Letunic I. Jensen L. J. Bork P. (2010). A side effect resource to capture phenotypic effects of drugs. Mol. Syst. Biol. 6, 343. 10.1038/msb.2009.98 20087340 PMC2824526

[B37] Liu Z. Shi Q. Ding D. Kelly R. Fang H. Tong W. (2011). Translating clinical findings into knowledge in drug safety evaluation–drug induced liver injury prediction system (DILIps). PLoS Comput. Biol. 7:e1002310. 10.1371/journal.pcbi.1002310 22194678 PMC3240589

[B38] Lounkine E. Keiser M. J. Whitebread S. Mikhailov D. Hamon J. Jenkins J. L.. (2012). Large-scale prediction and testing of drug activity on side-effect targets. Nature 486, 361–367. 10.1038/nature11159 22722194 PMC3383642

[B39] Melville P. Mooney R. J. (2003). Constructing diverse classifier ensembles using artificial training examples, in Proceedings of the 18th International Joint Conference on Artificial Intelligence (Acapulco: Morgan Kaufmann Publishers Inc.).

[B40] Munos B. (2009). Lessons from 60 years of pharmaceutical innovation. Nat. Rev. Drug Discov. 8, 959–968. 10.1038/nrd2961 19949401

[B41] Quinlan J. R. (1993). C4.5: Programs for Machine Learning. San Mateo, CA: Morgan Kaufmann Publishers.

[B42] Rattei T. Tischler P. Gotz S. Jehl M. A. Hoser J. Arnold R.. (2010). SIMAP–a comprehensive database of pre-calculated protein sequence similarities, domains, annotations and clusters. Nucleic Acids Res. 38, D223–D226. 10.1093/nar/gkp949 19906725 PMC2808863

[B43] Riss J. Cloyd J. Gates J. Collins S. (2008). Benzodiazepines in epilepsy: pharmacology and pharmacokinetics. Acta Neurol. Scand. 118, 69–86. 10.1111/j.1600-0404.2008.01004.x 18384456

[B44] Rodríguez J. J. Kuncheva L. I. (2006). Rotation forest: a new classifier ensemble method. IEEE Trans. Pattern Anal. Mach. Intell. 28, 1619–1630. 10.1109/TPAMI.2006.211 16986543

[B45] Roodhart J. M. Langenberg M. H. Witteveen E. Voest E. E. (2008). The molecular basis of class side effects due to treatment with inhibitors of the VEGF/VEGFR pathway. Curr. Clin. Pharmacol. 3, 132–143. 10.2174/157488408784293705 18690886

[B46] Scannell J. W. Blanckley A. Boldon H. Warrington B. (2012). Diagnosing the decline in pharmaceutical RandD efficiency. Nat. Rev. Drug Discov. 11, 191–200. 10.1038/nrd3681 22378269

[B47] Schaefer M. H. Fontaine J. F. Vinayagam A. Porras P. Wanker E. E. Andrade-Navarro M. A. (2012). HIPPIE: integrating protein interaction networks with experiment based quality scores. PLoS ONE 7:e31826. 10.1371/journal.pone.0031826 22348130 PMC3279424

[B48] Sing T. Sander O. Beerenwinkel N. Lengauer T. (2005). ROCR: visualizing classifier performance in R. Bioinformatics 21, 3940–3941. 10.1093/bioinformatics/bti623 16096348

[B49] Singhal S. Mehta J. Desikan R. Ayers D. Roberson P. Eddlemon P.. (1999). Antitumor activity of thalidomide in refractory multiple myeloma. N. Engl. J. Med. 341, 1565–1571. 10.1056/NEJM199911183412102 10564685

[B50] Stephens T. D. Bunde C. J. Fillmore B. J. (2000). Mechanism of action in thalidomide teratogenesis. Biochem. Pharmacol. 59, 1489–1499. 10.1016/S0006-2952(99)00388-3 10799645

[B51] Tomek I. (1976). 2 Modifications of Cnn. IEEE Trans. Syst. Man Cybern. 6, 769–772. 10.1109/TSMC.1976.4309452

[B52] Valavanis I. Spyrou G. Nikita K. (2010). A similarity network approach for the analysis and comparison of protein sequence/structure sets. J. Biomed. Inform. 43, 257–267. 10.1016/j.jbi.2010.01.005 20097308

[B53] Venkatesan K. Rual J. F. Vazquez A. Stelzl U. Lemmens I. Hirozane-Kishikawa T.. (2009). An empirical framework for binary interactome mapping. Nat. Methods 6, 83–90. 10.1038/nmeth.1280 19060904 PMC2872561

[B54] Verheul H. M. W. Panigrahy D. Yuan J. D'Amato R. J. (1999). Combination oral antiangiogenic therapy with thalidomide and sulindac inhibits tumour growth in rabbits. Br. J. Cancer 79, 114–118. 10.1038/sj.bjc.6690020 10408702 PMC2362163

[B55] Weston J. Elisseeff A. Zhou D. Leslie C. S. Noble W. S. (2004). Protein ranking: from local to global structure in the protein similarity network. Proc. Natl. Acad. Sci. U.S.A. 101, 6559–6563. 10.1073/pnas.0308067101 15087500 PMC404084

[B56] Whitehead T. A. Chevalier A. Song Y. Dreyfus C. Fleishman S. J. De Mattos C.. (2012). Optimization of affinity, specificity and function of designed influenza inhibitors using deep sequencing. Nat. Biotechnol. 30, 543–548. 10.1038/nbt.2214 22634563 PMC3638900

[B57] Wieseler B. Wolfram N. Mcgauran N. Kerekes M. F. Vervölgyi V. Kohlepp P.. (2013). Completeness of reporting of patient-relevant clinical trial outcomes: comparison of unpublished clinical study reports with publicly available data. PLoS Med. 10:e1001526. 10.1371/journal.pmed.1001526 24115912 PMC3793003

[B58] Wishart D. S. Knox C. Guo A. C. Shrivastava S. Hassanali M. Stothard P.. (2006). DrugBank: a comprehensive resource for in silico drug discovery and exploration. Nucleic Acids Res. 34, D668–D672. 10.1093/nar/gkj067 16381955 PMC1347430

[B59] Witten I. H. Frank E. Hall M. A. (2011). Data Mining: Practical Machine Learning Tools and Techniques. Burlington, MA: Morgan Kaufmann.

[B60] Xie L. Li J. Xie L. Bourne P. E. (2009). Drug discovery using chemical systems biology: identification of the protein-ligand binding network to explain the side effects of cetp inhibitors. PLoS Comput. Biol. 5:e1000387. 10.1371/journal.pcbi.1000387 19436720 PMC2676506

[B61] Yamanishi Y. Araki M. Gutteridge A. Honda W. Kanehisa M. (2008). Prediction of drug-target interaction networks from the integration of chemical and genomic spaces. Bioinformatics 24, i232–i240. 10.1093/bioinformatics/btn162 18586719 PMC2718640

[B62] Yildirim M. A. Goh K. I. Cusick M. E. Barabási A. L. Vidal M. (2007). Drug-target network. Nat. Biotechnol. 25, 1119–1126. 10.1038/nbt1338 17921997

[B63] Zhang Z. Grigorov M. G. (2006). Similarity networks of protein binding sites. Proteins 62, 470–478. 10.1002/prot.20752 16299776

[B64] Zhu F. Shi Z. Qin C. Tao L. Liu X. Xu F.. (2012). Therapeutic target database update 2012: a resource for facilitating target-oriented drug discovery. Nucleic Acids Res. 40, D1128–D1136. 10.1093/nar/gkr797 21948793 PMC3245130

