# Supplementary figures and images for: Identifying problematic drugs based on the characteristics of their targets
Source: Front Pharmacol. 2015 Sep 1;6:186. doi: 10.3389/fphar.2015.00186 (PMC4555035; doi:10.3389/fphar.2015.00186)

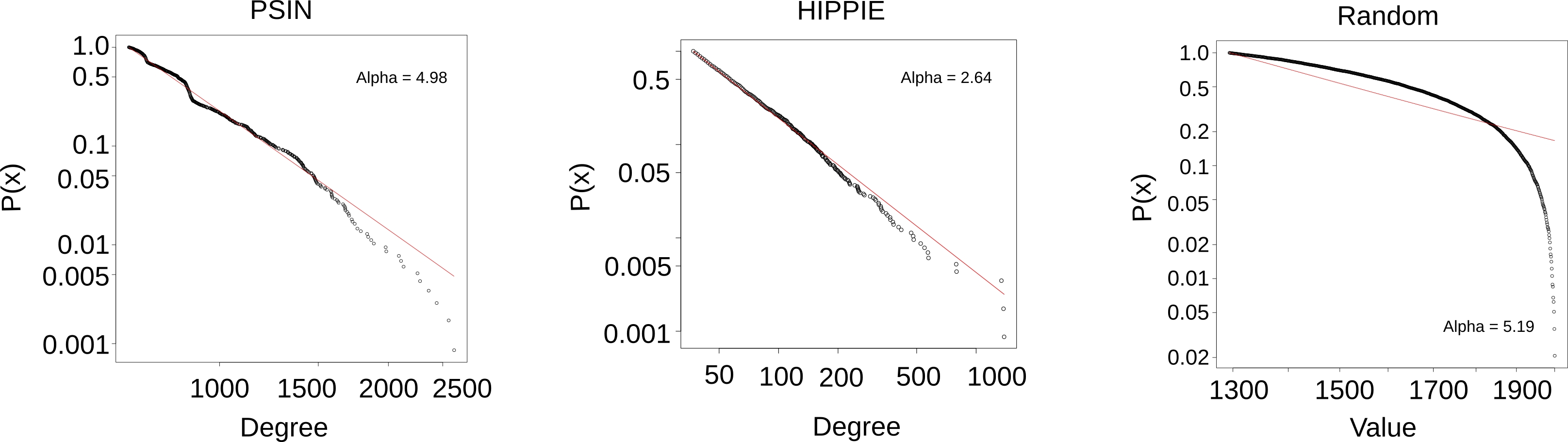

Supplement: Supplementary Figure S1 — We verified that the PSIN degree distribution could be fitted in the power-law. The figure depicts the degree distribution (black circles) and the curve-fit (in red), with the modifying exponent parameter indicated in the figure. The fit is satisfactory for most nodes, but those with a higher degree have some deviation from the fitted curve. Additionally, we can observe the degree distribution for the HIPPIE database, and for a sequence of 15,000 random numbers chosen with replacement from the interval [1, 2000]. [file DataSheet1.ZIP › Supplementary/S1.JPEG]

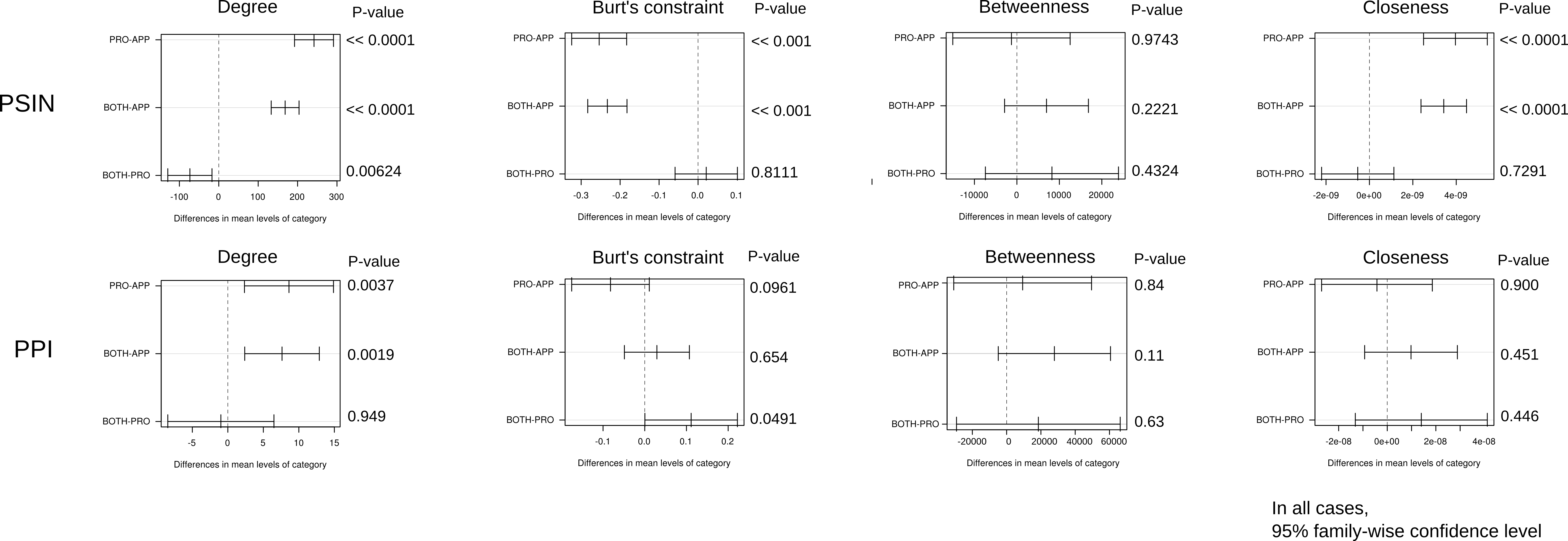

Supplement: Supplementary Figure S1 — We verified that the PSIN degree distribution could be fitted in the power-law. The figure depicts the degree distribution (black circles) and the curve-fit (in red), with the modifying exponent parameter indicated in the figure. The fit is satisfactory for most nodes, but those with a higher degree have some deviation from the fitted curve. Additionally, we can observe the degree distribution for the HIPPIE database, and for a sequence of 15,000 random numbers chosen with replacement from the interval [1, 2000]. [file DataSheet1.ZIP › Supplementary/S2.JPEG]

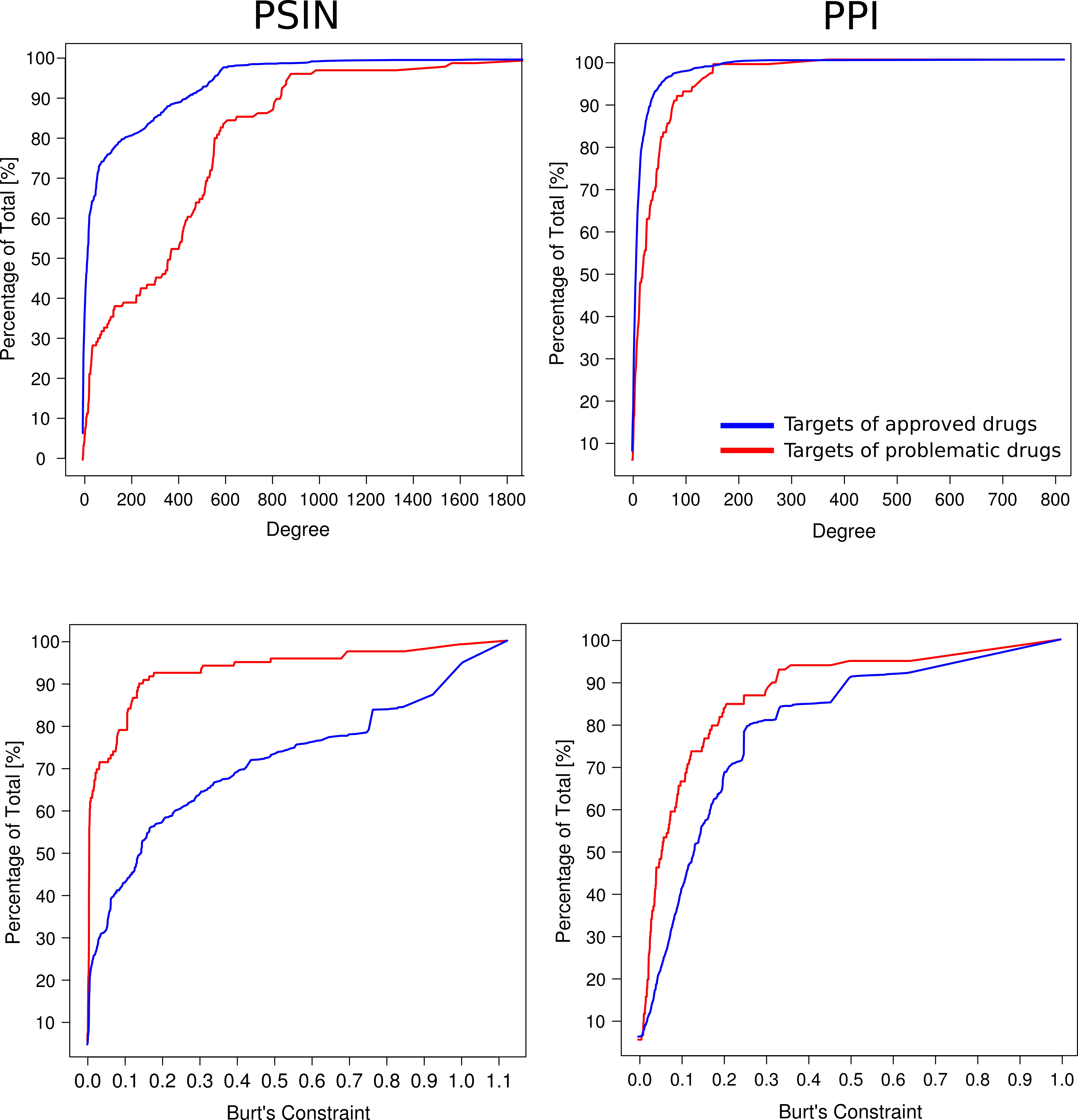

Supplement: Supplementary Figure S1 — We verified that the PSIN degree distribution could be fitted in the power-law. The figure depicts the degree distribution (black circles) and the curve-fit (in red), with the modifying exponent parameter indicated in the figure. The fit is satisfactory for most nodes, but those with a higher degree have some deviation from the fitted curve. Additionally, we can observe the degree distribution for the HIPPIE database, and for a sequence of 15,000 random numbers chosen with replacement from the interval [1, 2000]. [file DataSheet1.ZIP › Supplementary/S3.JPEG]

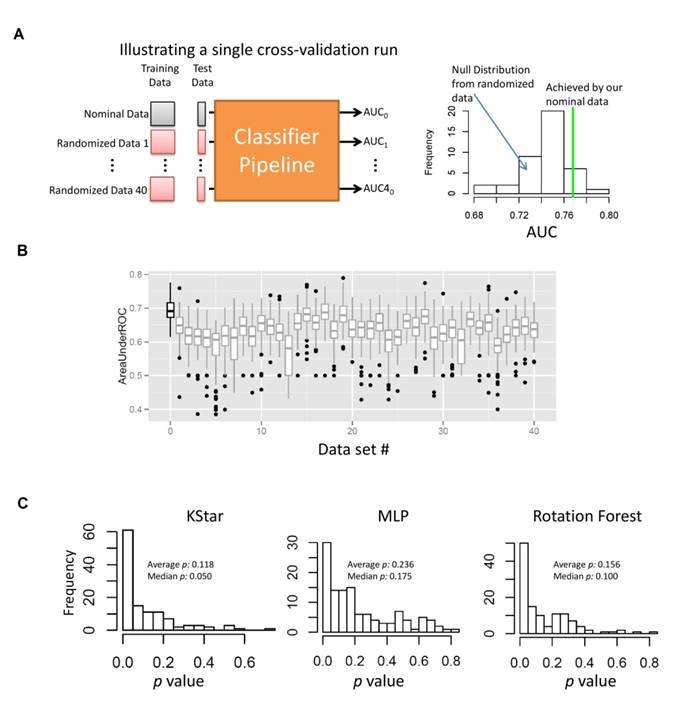

Supplement: Supplementary Figure S1 — We verified that the PSIN degree distribution could be fitted in the power-law. The figure depicts the degree distribution (black circles) and the curve-fit (in red), with the modifying exponent parameter indicated in the figure. The fit is satisfactory for most nodes, but those with a higher degree have some deviation from the fitted curve. Additionally, we can observe the degree distribution for the HIPPIE database, and for a sequence of 15,000 random numbers chosen with replacement from the interval [1, 2000]. [file DataSheet1.ZIP › Supplementary/S5.JPEG]

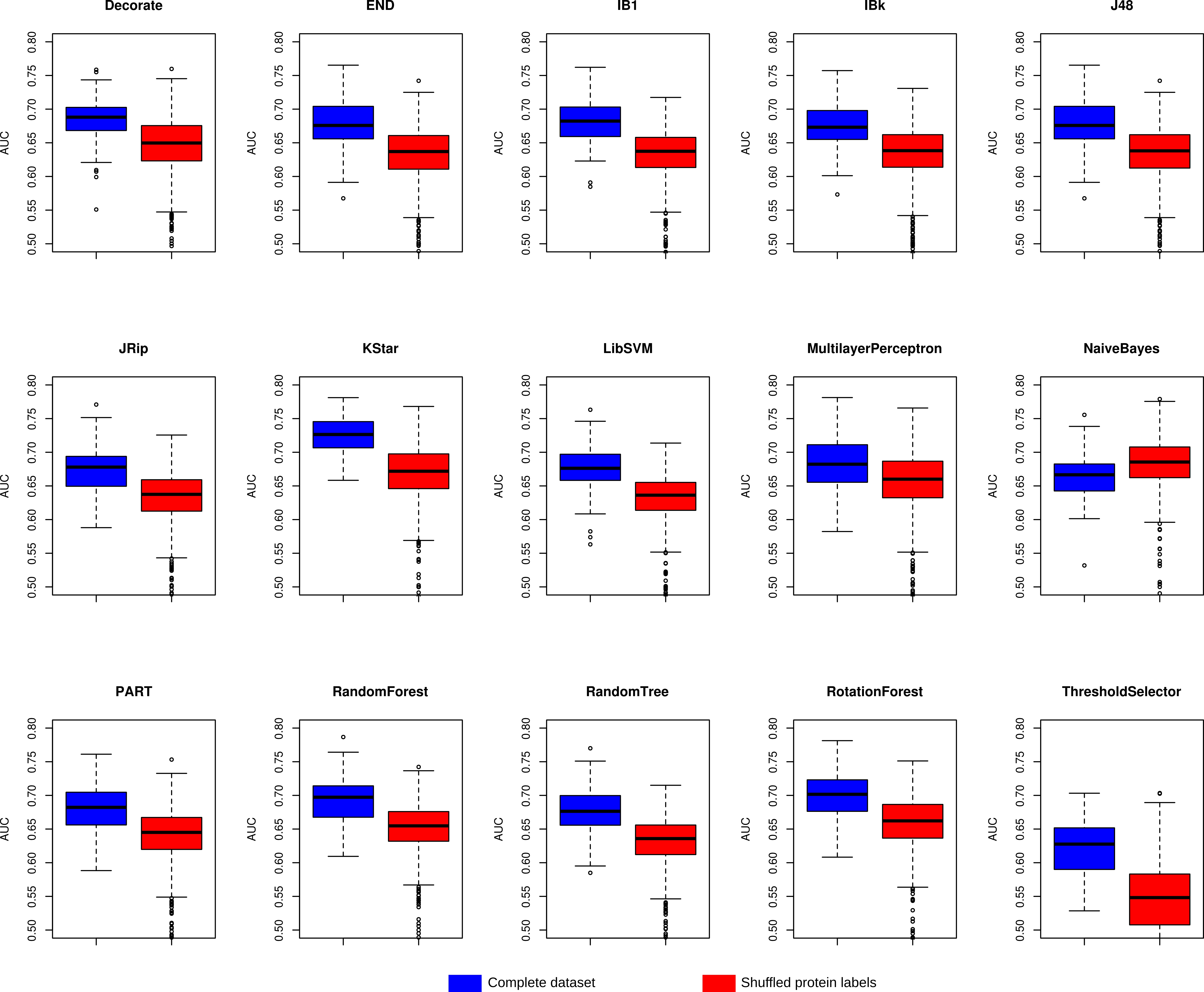

Supplement: Supplementary Figure S1 — We verified that the PSIN degree distribution could be fitted in the power-law. The figure depicts the degree distribution (black circles) and the curve-fit (in red), with the modifying exponent parameter indicated in the figure. The fit is satisfactory for most nodes, but those with a higher degree have some deviation from the fitted curve. Additionally, we can observe the degree distribution for the HIPPIE database, and for a sequence of 15,000 random numbers chosen with replacement from the interval [1, 2000]. [file DataSheet1.ZIP › Supplementary/S6.JPEG]

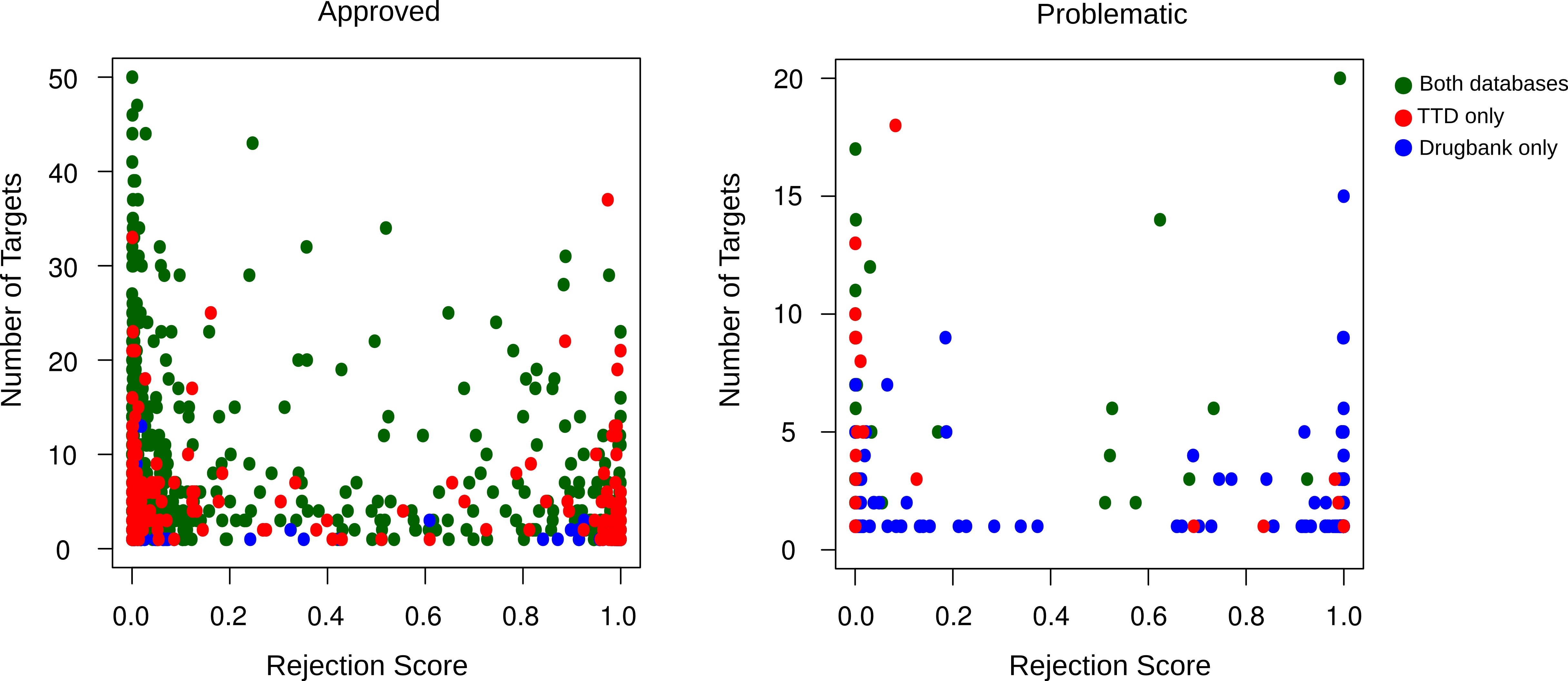

Supplement: Supplementary Figure S1 — We verified that the PSIN degree distribution could be fitted in the power-law. The figure depicts the degree distribution (black circles) and the curve-fit (in red), with the modifying exponent parameter indicated in the figure. The fit is satisfactory for most nodes, but those with a higher degree have some deviation from the fitted curve. Additionally, we can observe the degree distribution for the HIPPIE database, and for a sequence of 15,000 random numbers chosen with replacement from the interval [1, 2000]. [file DataSheet1.ZIP › Supplementary/S7.JPEG]
